# Geochemical and mineralogical evidence that Rodinian assembly was unique

**DOI:** 10.1038/s41467-017-02095-x

**Published:** 2017-12-05

**Authors:** Chao Liu, Andrew H. Knoll, Robert M. Hazen

**Affiliations:** 1 Geophysical Laboratory, Carnegie Institution for Science, Washington, DC 20015 USA; 2 Department of Earth and Planetary Sciences, Harvard University, Cambridge, MA 02138 USA

## Abstract

The mineralogy and geochemistry associated with Rodinian assembly (~1.3–0.9 Ga) are significantly different from those of other supercontinents. Compared to other supercontinents, relatively more Nb-bearing minerals, Y-bearing minerals, and zircons formed during Rodinian assembly, with corresponding enrichments of Nb, Y, and Zr concentrations in igneous rocks. By contrast, minerals bearing many other elements (e.g., Ni, Co, Au, Se, and platinum group elements) are significantly less abundant, without corresponding depletion of Ni and Co concentrations in igneous rocks. Here we suggest that the Nb, Y, and Zr enrichments in igneous rocks and relatively more occurrences of corresponding Nb-bearing minerals, Y-bearing minerals, and zircons result from significant non-arc magmatism during the mid-Proterozoic, while fewer occurrences of many other minerals suggest enhanced erosion of Rodinian volcanic arcs and orogens. The prolonged, extrovert assembly of Rodinia from thickened mid-Proterozoic continental crust via two-sided subduction can account for both the prevalence of non-arc magmatism and the enhanced erosion.

## Abstract (web summary)

The supercontinent Rodinia has been hypothesised to have formed in a different manner from other supercontinents. Here, the authors report geochemical and mineralogical evidence for prevalence of non-arc magmatism and enhanced erosion of volcanic arcs and orogens during Rodinian assembly.

## Introduction

Supercontinents have assembled and disperzed repeatedly since the late Archean Eon, recorded most conspicuously by the age frequency distribution of detrital zircons^1^. Episodes of supercontinent amalgamation share a number of tectonic and geochemical features^2^; however, increasing evidence suggests that no two supercontinents formed in quite the same manner^3,4,5^. In particular, it has been hypothesized that the supercontinent Rodinia differs distinctly from other supercontinents^3^; Rodinia was assembled through a series of accretionary and collisional events between 1.3 and 0.9 billion years ago^6^; endured through the late stages of Earth’s middle age^7^; and broke apart in association with pronounced perturbations to the carbon cycle, global glaciations^8^, and the rise of complex multicellular life^9^. Similar to those of other supercontinents, Rodinian assembly (RA) is marked by a peak in the abundance of detrital zircons with contemporary ages, archiving an integrated result of crustal generation and preservation^3,10^ or punctuated crustal growth^11,12^. Previous studies proposed that Rodinia stands out from other supercontinents in many aspects, such as enhanced anorogenic magmatism, deficiency in continental margins and collisional belts, and dearth in ore deposits and minerals of precious metals, Hg, and other elements^7,13–17^. Many of these proposed aspects, however, are based on regional or outdated geologic and geochemical databases with limited data^15,18,19^. Recently, rapidly expanding global databases of geochemistry, mineralogy, and stratigraphy have begun to facilitate studies of Earth as a system, with emphasis on our planet’s evolution through time^20–22^. In this study, we compile and analyze existing global databases of minerals (data from rruff.info/ima) and igneous geochemistry (data from earthchem.org) through time to test the extent to which Rodinia is geochemically and mineralogically distinct from other supercontinents, and to explore possible reasons underlying observed differences.

Our results indicate that niobium (Nb), yttrium (Y), and zirconium (Zr) concentrations in igneous rocks formed during RA are statistically higher, coupled with more abundances of Nb-bearing and Y-bearing minerals, but many other minerals are less abundant during RA than during assembly of other supercontinents. Such anomalies can be explained by prevalence of non-arc magmatism and enhanced erosion during RA.

## Results

### Mineral data

**Fig. 1:**
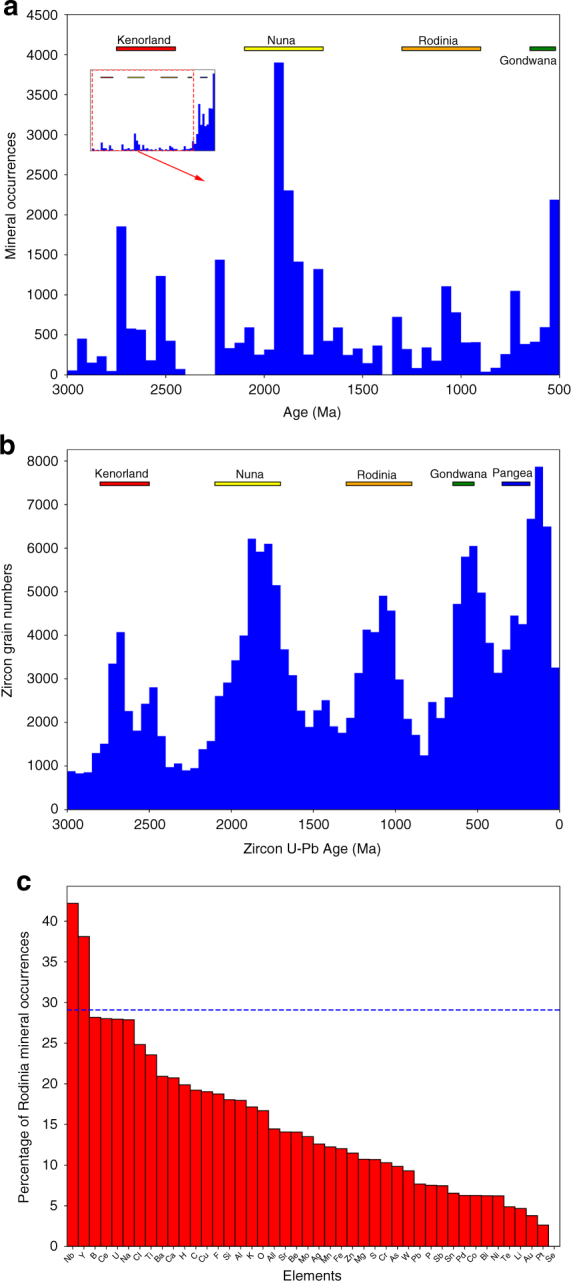
Temporal distribution of global high-T minerals and detrital zircons. Igneous, metamorphic, and hydrothermal minerals (**a**) and detrital zircons (**b**) through the last 3.0 Ga, with a bin size of 50 Ma. Mineral occurrence is defined in the Methods section. Detrital zircon distribution data is based on ref. ^24^. In spite of the significant preservation bias toward the present, high-T minerals are generally enriched during supercontinental assemblies, similar to detrital zircons. During Rodinian assembly, high-T minerals are relatively less abundant compared to other supercontinents Panel **a** displays 3000-500 Ma; inset displays 3000-0 Ma. **c** A survey based on mineral chemistry showing relative abundances of minerals containing different elements. *Y*-axis is defined as the percentage of entry numbers of specific minerals occurring during RA relative to total entry numbers of those minerals occurring during assemblies of all pre-Pangia supercontinents (dashed blue lines: the percentage of detrital zircon). Except for Nb-bearing and Y-bearing minerals, most minerals are relatively depleted compared to detrital zircon during Rodinia assembly

From rruff.info/ima, we compiled 108,857 age-locality records of high-temperature (high-T, i.e., igneous, metamorphic, and hydrothermal) minerals for which ages are well constrained from radiometric dating of corresponding magmatic, metamorphic, or hydrothermal events. Spatially, these minerals are distributed globally (Supplementary information; Supplementary Fig. 1). Temporally, despite a preservational bias toward deposits of the Phanerozoic Eon, the high-T minerals exhibit ages more commonly associated with supercontinent assembly (Fig. 1), similar to detrital zircons^23,24^. This similarity, however, breaks down during RA; the abundance of detrital zircons exhibit one of the strongest peaks observed throughout Earth history, but occurrences of high-T minerals in total are much less pronounced (Fig. 1). Analysis of the high-T mineral data based on mineral chemistry reveals that only a few minerals, including Nb-bearing and Y-bearing minerals, are relatively more abundant than zircons during RA, and that most high-T minerals, especially minerals bearing selenium (Se), gold (Au), nickel (Ni), cobalt (Co), and platinum group elements (PGE), are significantly less abundant at the same time (Fig. 1).

### Geochemical data

**Fig. 2:**
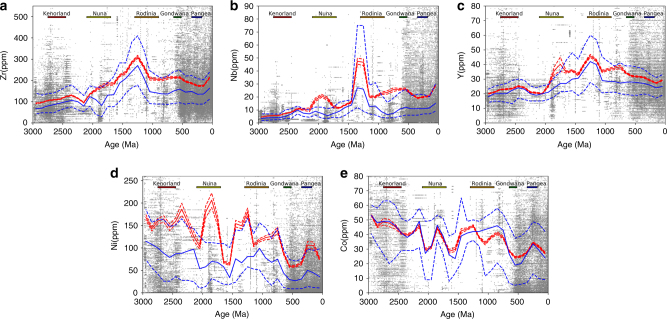
Trace element concentration. (**a**, Zr; **b**, Nb; **c**, Y; **d**, Ni; and **e**, Co) in global igneous rocks through the last 3.0 Ga. Zr, Nb, and Y exhibit the highest values during and immediately before Rodinian assembly, while Ni and Co show no depletions at the same time. The gray filled circles are data resampled from Earthchem with bootstrap resampling. Moving averages and medians of samples within ±100 Ma bin size are calculated for each 100 Ma. Red solid line: average; Red dashed lines: 95% confidence interval of the moving average; Blue solid line: median; Blue dashed lines: the lower (25%) and upper (75%) quantiles

We compiled whole-rock chemical analyses of dated igneous rocks from http://www.earthchem.org/portal, including concentration data on 129,161 samples for Zr, 105,045 for Nb, 121,373 for Y, 77,835 for Co, and 82,611 for Ni—all are associated with SiO_2_ content (wt%) and modern geographic coordinates. Similar to our mineral data, the extracted geochemical data are globally distributed (Supplementary Fig. 1). During RA, multiple statistics of Nb, Y, and Zr concentrations in igneous rocks exhibit the highest values in the last 3.0 Ga (Fig. 2), significantly higher than the values during assembly of any other supercontinent (Table 1). Such geochemical enrichments are statistically significant in both mafic and felsic igneous rocks (Table 1), consistent with previous reports of anomalously high Zr in Laurentian granitoids^18^ and igneous samples from a smaller, older-version Earthchem database^19^. Unlike Nb, Y, and Zr, neither enrichments nor depletions are observed for Ni and Co concentrations in igneous rocks formed during RA (Fig. 2).

**Table 1:** Results of two-sample Welch’s *t*-tests of Zr, Nb, and Y concentrations between igneous rocks formed during Rodinian assembly and those formed during assemblies of other supercontinents

	All		Mafic		Felsic	
	*t*-statistic	*p*-value	*t*-statistic	*p*-value	*t*-statistic	*p*-value
Zr						
Rodinia–Kenorland	54.86	0	52.01	0	13.2	3.07E−35
Rodinia–Nuna	24.63	1.10E−128	25.9	1.51E−137	5.15	3.60E−07
Rodinia–Gondwana	2.12	0.03	7.38	3.02E−13	2.42	0.02
Rodinia–Pangea	26.47	1.67E−149	23.16	1.86E−113	6.81	1.77E−11
Nb						
Rodinia–Kenorland	46.08	0	22.58	2.72E−101	19.74	6.15E−66
Rodinia–Nuna	14.36	3.37E−46	8.03	1.51E−15	4.16	3.51E−05
Rodinia–Gondwana	4.33	1.52E−05	−0.13	0.88	3.88	0.0001
Rodinia–Pangea	15.24	7.11E−52	2.05	0.04	1.7	0.09
Y						
Rodinia–Kenorland	44.02	0	40.3	5.25E−304	23.38	1.91E−96
Rodinia–Nuna	19.59	1.76E−83	24.49	1.57E−123	6.99	5.24E−12
Rodinia–Gondwana	13.35	2.59E−40	10.62	5.03E−25	9.83	8.69E−22
Rodinia–Pangea	24.1	6.43E−125	25.07	1.07E−132	7.65	3.49E−14

The *t* statistics between Rodinia and another supercontinent are mostly positive with corresponding *p*-values <0.05, suggesting statistically significant enrichments of Zr, Nb, and Y for Rodinian igneous rocks

## Discussion

**Fig. 3:**
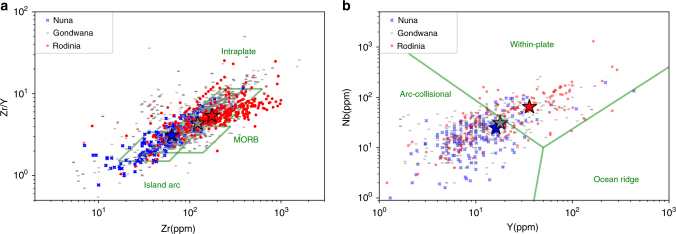
Tectonic discrimination diagrams. Discrimination diagrams^25,26^ for mafic (**a**) and felsic (**b**) rocks formed during assemblies of Nuna (blue, x), Rodinia (red filled circle), and Gondwana (gray, -) based on concentrations of immobile trace elements. Average values for each supercontinent are plotted as filled stars. “Within-plate/Intraplate” geochemical signatures are dominant for igneous rocks formed during Rodinia assembly, whereas arc-collisional magmatism is relatively more significant for Nuna and Gondwana

Compared to other supercontinental assemblies, the enrichments of Nb, Y, and Zr in igneous rocks and more abundant Nb-bearing, Y-bearing minerals, and detrital zircons strongly suggest distinctive tectonics during RA, leading to unique patterns of magmatism and mineralization. For both mafic and felsic igneous rocks, tectonic discrimination^25,26^ based on the immobile trace elements (Fig. 3) implies that geochemical signatures of “within-plate” magmatism prevail during RA, whereas island arc and collisional magmatism is more, or at least equally, significant during the assembly of other supercontinents (e.g., Nuna, Gondwana). Unlike “within-plate” magmatic rocks normally discovered in intraplate settings, these rocks formed during RA are associated with not only intracontinental rifting^27,28^, but also back-arc settings^29^, and zones of orogenic distension/exhumation during episodic collisional hiatus^30–32^, as long as the tectonic setting is extensional. Such widespread extensional magmatism can be attributed to enhanced asthenosphere–lithosphere interactions^27,30,33,34^, possibly involving a warmer mantle^35^ and/or a thicker continental crust^36^ than at present. It has been speculated that there was a large-scale mantle thermal anomaly^35,37^, possibly due to thermal blanketing and/or heat down-welling of the mantle beneath the long-lived supercontinent Nuna^38,39^. Alternatively, it has been proposed that continental lithosphere was strong enough to be thickened^36^ and to support the emplacement of large plutons into the crust, yet the underlying mantle was still warm enough to result in widespread melting of the lower thickened continental crust^7,13,40^.

Many types of magmatic rocks that were formed during RA, including massif anorthosites^41,42^, A-type granitoids^18,43–45^, and NYF (Nb, Y, and F enriched)-type pegmatites^31,46–48^, are enriched in Zr, Nb, and Y relative to arc magmatic rocks^18,34,47^. In arc magmas, these elements are scavenged by interaction with depleted mantle peridotite during subduction^49,50^, while this interaction was mitigated during enhanced asthenosphere–lithosphere interaction^30,33^ or even circumvented during the melting of continental crust^40^, due to a warmer mantle^35^ and/or a thickened continental crust^36^ during RA. Of all the non-arc magmatic rocks formed during RA, NYF-type pegmatites are the most enriched in Nb and Y, together with fluorine (F). The enrichment of Nb and Y is amplified by the strong partitioning of Nb and Y into F-rich fluids and melts^51–54^, and F has been proposed to be sourced from decomposing F-rich biotite and amphibole during crustal anatexis^31,55,56^. As a result, NYF-type pegmatites bear a plethora of rare Nb-bearing and Y-bearing minerals, consistent with the observed Nb and Y mineral enrichment during RA.

Unlike the coupled mineral and geochemical enrichments of Nb, Y, and Zr during RA, the significant depletions in the abundances of many other minerals (e.g., Se, Au, Ni, Co, and PGE minerals) are not accompanied by any corresponding elemental depletion in igneous rocks (Fig. 2). Rather, diminished mineralization is consistent with a previously reported dearth of ore deposits enriched in these minerals^7^, including volcanic-hosted massive sulfides (VHMS), porphyry-related metals, and Au deposits^14,16^. Why these ore deposits are scarce is still an open question. Although many other processes are possible, currently proposed mechanisms include rarity of reduced ore fluids in a relatively oxidized Mesoproterozoic atmosphere^57^, or poor preservation because of erosion^14,16^. We speculate that the former is less likely, not only because it is at odds with reported low atmospheric O_2_^58,59^ during the Mesoproterozoic Era, but also because of the observed enrichment of these deposits during Nuna assembly when atmospheric O_2_, at best, was similarly low^57^. Instead, our observation of relatively less Nb, Y, Zr-depleted, arc magmatic samples during RA (Figs. 2, 3) favors enhanced tectonic erosion of active margins, where most of these ore deposits occur or are preferentially preserved^14,16^. Preferential erosion might be considered a limiting preservational bias, but in this case we argue that it reflects tectonic processes specifically associated with RA.

Both pre-collisional and orogenic erosion events might have contributed to the observed dearth in mineral and ore deposits during RA. Pre-collisional erosion during RA may have been more significant than that associated with other supercontinents, because Rodinia accretion is proposed to have been prolonged, and extrovert^60^ via two-sided subduction^61,62^. This tectonic context may have doomed the preservation of VHMS deposits, which requires rapid accretion of continental margins^14^. In addition, many Rodinian orogens (e.g., Grenville, Sveconorwegian, Namaqualand–Natal) exhibit episodic collisions with distension intervals^6,63^, which could facilitate orogenic erosion. Indeed, deep erosion has been observed for the Grenville^63,64^ and the Sveconorwegian orogens^65,66^, which constitute the main collisional suture of Rodinia^6^. Enhanced orogenic erosion is consistent with possible development of large-scale river systems and massive Grenvillian fluvial sediments^67,68^. Removal of the shallow part of the orogens could account for the absence of Au deposits during RA, which normally occur at <10 km depth^16^.

In general, the observed mineral enrichments and depletions during RA (Fig. 1) are an integrated result of mineral crystallization and preservation, instead of purely mineral genesis. The interplay of formation and destruction can also account for the temporal distribution of global detrital zircons^3^. During RA, a unique tectonic setting led to non-arc magmatism and enhanced erosion, which in turn resulted in geochemical, mineral, and ore enrichments and depletions that established Rodina as distinct among supercontinental events.

## Methods

### Database description and compilation

Compilation of the data, including data query, data filtering, and data resampling, is performed with Pandas 0.21.0 implemented in Python 2.7.11, in which randomness is simulated with Mersenne Twister pseudo-random number generator.

Data on mineral ages, localities, and coordinates are extracted from rruff.info/ima (as of May 2016), developed at University of Arizona. It is a relational database, with attributes including mineral name, structure, chemical formula, locality name, coordinates, paragenetic mode, and age (if available). Most of the entries are sourced from mindat.org, in which the localities are typically defined on a mine level, distributed at least 5 km apart. In addition, we have added new entries into the rruff database through brute-force search in scientific publications. In this paper, we queried only high-temperature (high-T, e.g., igneous, metamorphic, and hydrothermal) minerals, of which the ages are well constrained from dating corresponding magmatic, metamorphic, or hydrothermal events, for a total of 108,857 entries.

The high-T mineral entries are further queried by mineral chemistry to investigate occurrences of specific minerals in different geologic time. Queries based on mineral chemistry reveal that the degree of enrichment vary for different species during RA. We compiled the queries of elements to show that Nb and Y minerals are enriched, while most others, especially Se, Ni, Co, Au, and PGE elements, are relatively depleted during RA (Fig. 1). Note that we only include elements that make up minerals with at least 20 occurrences during assembly of each supercontinent to be statistically significant.

Geochemical data of igneous rocks are extracted from http://www.earthchem.org/portal (as of April 2017), which is a portal of multiple databases including the Petrological Database (PetDB; http://www.earthchem.org/petdb), North American Volcanic and Intrusive Rock Database (NAVDAT; http://www.navdat.org), the Geochemistry of Rocks of the Oceans and Continents database (GEOROC; http://georoc.mpch-mainz.gwdg.de/georoc), and the U.S. Geological Survey database (USGS; https://mrdata.usgs.gov/geochem/). It is also a relational database, with attributes including sample ID, rock type, major element concentrations, trace element concentrations, coordinates, ages, etc. We compiled concentrations in igneous samples of 129,161 Zr; 105,045 Nb; 121,373 Y; 77,835 Co; and 82,611 Ni whole-rock concentrations, all of which are dated, and associated with reported SiO_2_ content (wt%). We also tried to compile concentrations of PGE and Au, but the sample sizes are usually too small (<5000) to be statistically significant. In addition, we included ~50 data points of Zr, Nb, and Y of igneous rocks^19^ missing from EarthChem. The compiled data are further filtered to select only samples of ages between 0 and 3000 Ma, with age uncertainties <±200 Ma, and with legitimate geographic (latitudes within ±90°, longitudes within ±180°).

### Resampling

Bootstrap resampling was performed to minimize spatial and temporal sampling bias^21^. Sample weights were assigned to be inversely dependent on spatiotemporal sample density, according to the relationship

1 $$W_i \propto 1{\mathrm{/}}\mathop {\sum}\nolimits_{j = 1}^n {\left( {\frac{1}{{1 + {\left( {\left( {z_i - z_j} \right){\mathrm{/}}a} \right)}^2}} + \frac{1}{{1 + {\left( {\left( {t_i - t_j} \right){\mathrm{/}}b} \right)}^2}}} \right)}$$

where *n* is the number of samples in the database, *z* is spatial location, *t* is age of the rock, and *a* and *b* are normalization coefficients of 1.8 arc degrees (200 km) and 38 Myr, respectively. After calculation of weight *W*_*i*_ for each sample *i* in the database, bootstrap resampling was carried out by random selection of data points based on their weights, i.e., data with larger weights have higher chance to be selected. For each selected data, the synthetic data were drawn from a Gaussian distribution with a mean equal to the original value of the data point and standard deviation equal to the estimated 1*σ* uncertainty of the data point. It is shown that the analysis is insensitive to the resampling size. We built the resampling data set to a size identical to the original database.

### Statistical test and tectonic discrimination

The resampled data are plotted with *a* ± 100 Ma bin size at a frequency of 100 Ma (Fig. 2). The apparent enrichments of Zr, Nb, and Y concentrations during RA in Fig. 2 are examined as follows. First, trace metal concentrations during the assemblies of Nuna, Rodinia, and Gondwana share similar distribution patterns, ruling out the possibility that Rodinian enrichments are caused by outliers. In addition, two-sample Welch’s *t*-tests (Table 1) demonstrate that average Zr, Nb, and Y concentrations are generally higher during RA than other supercontinents (*t* > 0 and *p* < 0.05). What is more, this enrichment is statistically significant for both mafic (SiO_2_ 43–51 wt%) and felsic (SiO_2_ 62–73 wt%) samples in general. The *t*-test is performed with Scipy 0.19.0 implemented in Python 2.7.11.

Tectonic discrimination diagrams (Fig. 3) are plotted based on immobile trace elements (Zr, Nb, Y) in igneous rocks^25,26^. Such tectonic discrimination diagrams should be used with caution, especially when the rocks have a small sample size or are of limited spatial and temporal distribution^69,70^. Nevertheless, rocks in this study are sampled globally, with age ranges of several hundred million years, and sample sizes of several thousand for each supercontinent assembly. Therefore, the difference observed for immobile trace elements of different supercontinent assemblies suggests unequal tectonic settings.

### Data availability

All data analyzed in this study are downloaded from open source databases rruff.info/ima and http://www.earthchem.org/portal. Python codes used to analyze these data are available upon request by e-mailing cliu@carnegiescience.edu.

## Electronic supplementary material

Supplementary Information
