# Supplementary material for: Geochemical and mineralogical evidence that Rodinian assembly was unique
Source: Nat Commun. 2017 Dec 5;8:1950. doi: 10.1038/s41467-017-02095-x (PMC5717144; doi:10.1038/s41467-017-02095-x)

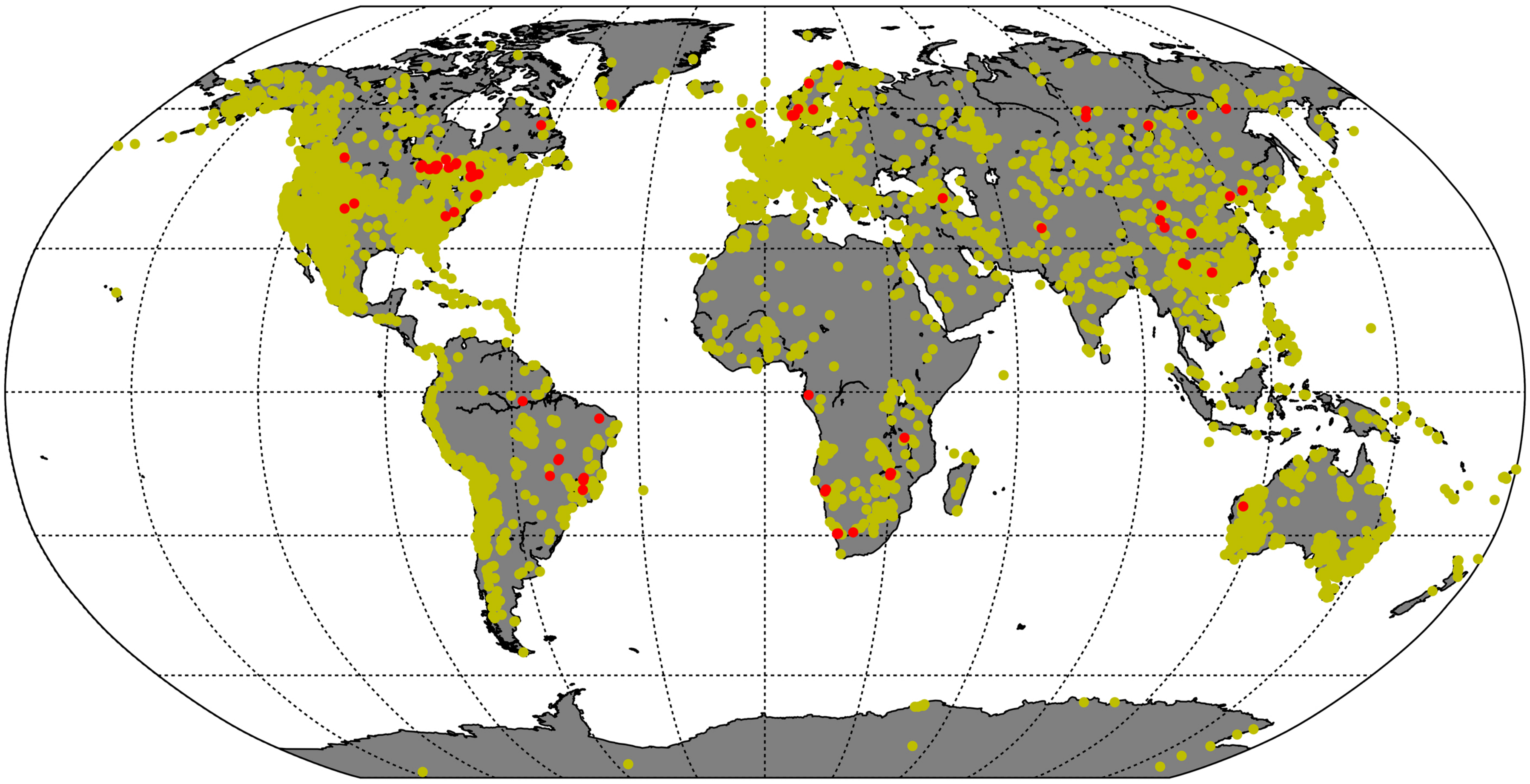

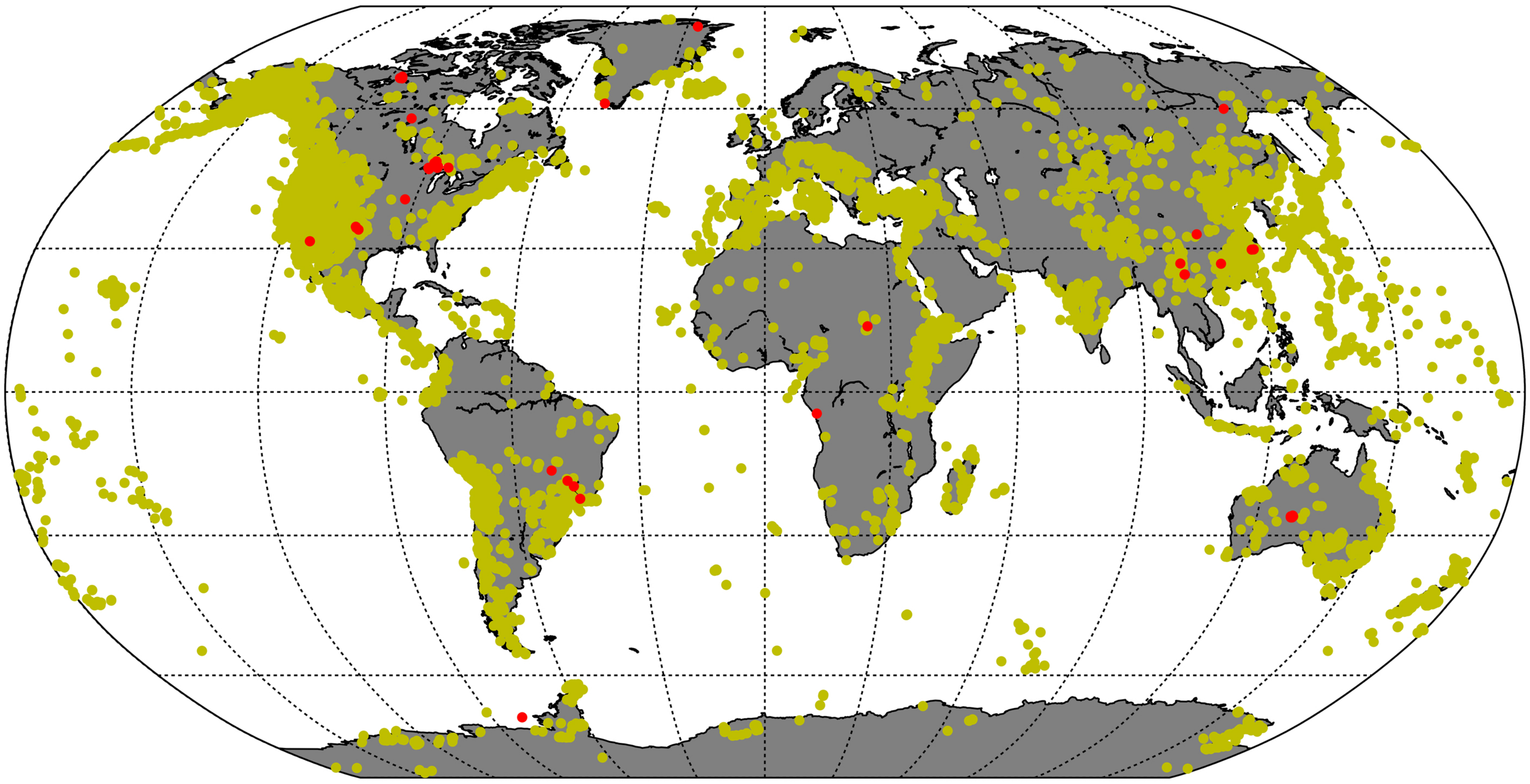

Supplementary Figure 1. Spatial distribution of high-T minerals (upper) and geochemical (lower) data. Both are globally distributed. Yellow dots: Coordinates of all dated data; Red dots: Coordinates of all dated data with ages between 1.3-0.9 Ga.

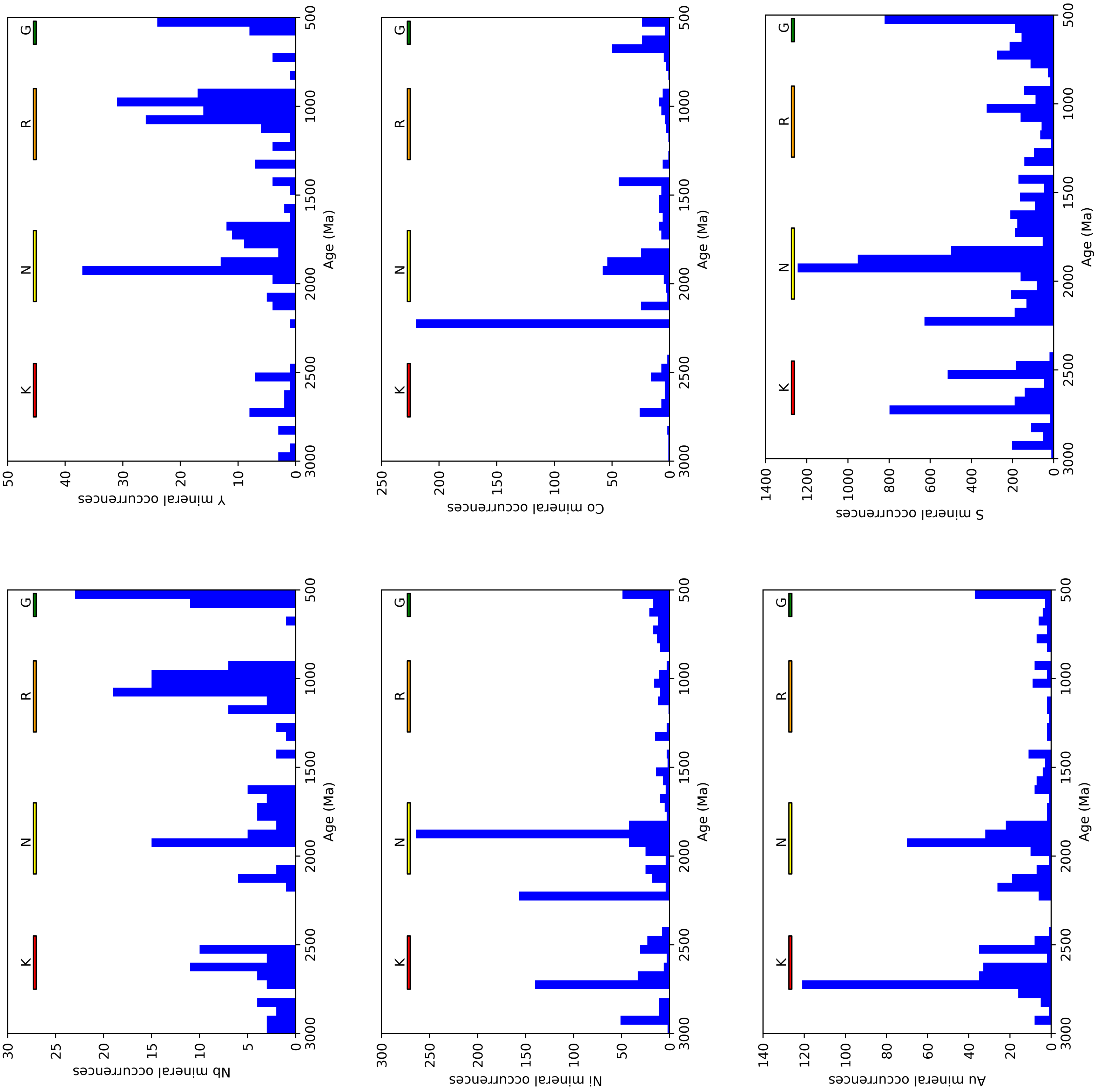

Supplementary Figure 2. Temporal distribution of selected minerals from 3.0 Ga to 0.5 Ga, with a bin size of 50 Ma. Nb, Y minerals are significant enriched, while most other species, including Au, Ni, Co, S minerals, do not exhibit prominent enrichment during Rodinian assembly.

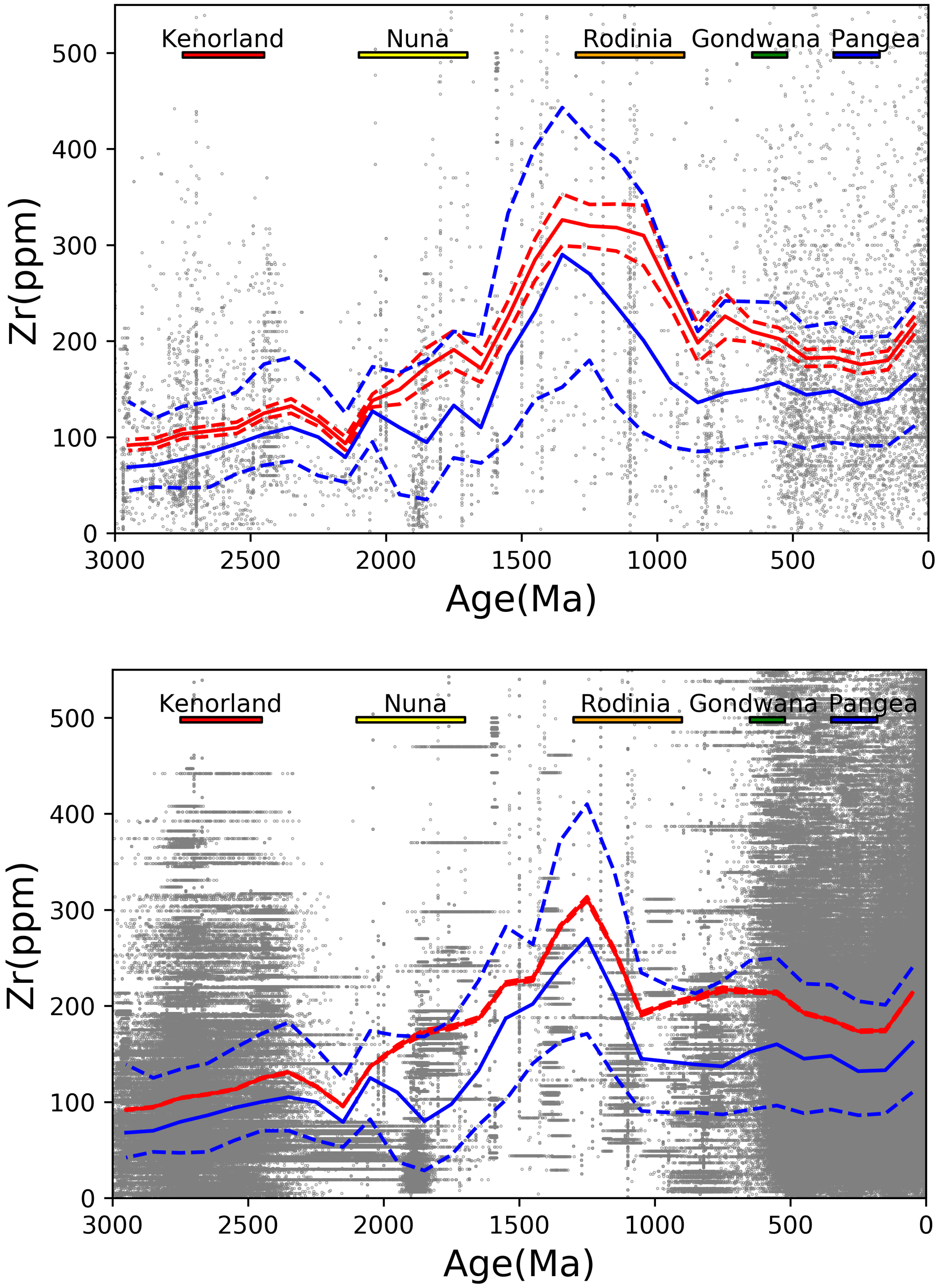

Supplementary Figure 3. Sensitivity test of sampling size in the bootstrap resampling of the Earthchem geochemical data, with the upper panel of 10%, and the lower panel of 10 times of the original sample size. Compared to the same-size resampling, different sampling sizes do not significantly shift values of the statistics.

Supplementary Figure 4. Distributions of Zr, Nb, Y concentrations of igneous rocks formed during assemblies of different supercontinents. All of them exhibit similar distribution patterns, i.e., a log-normal distribution.

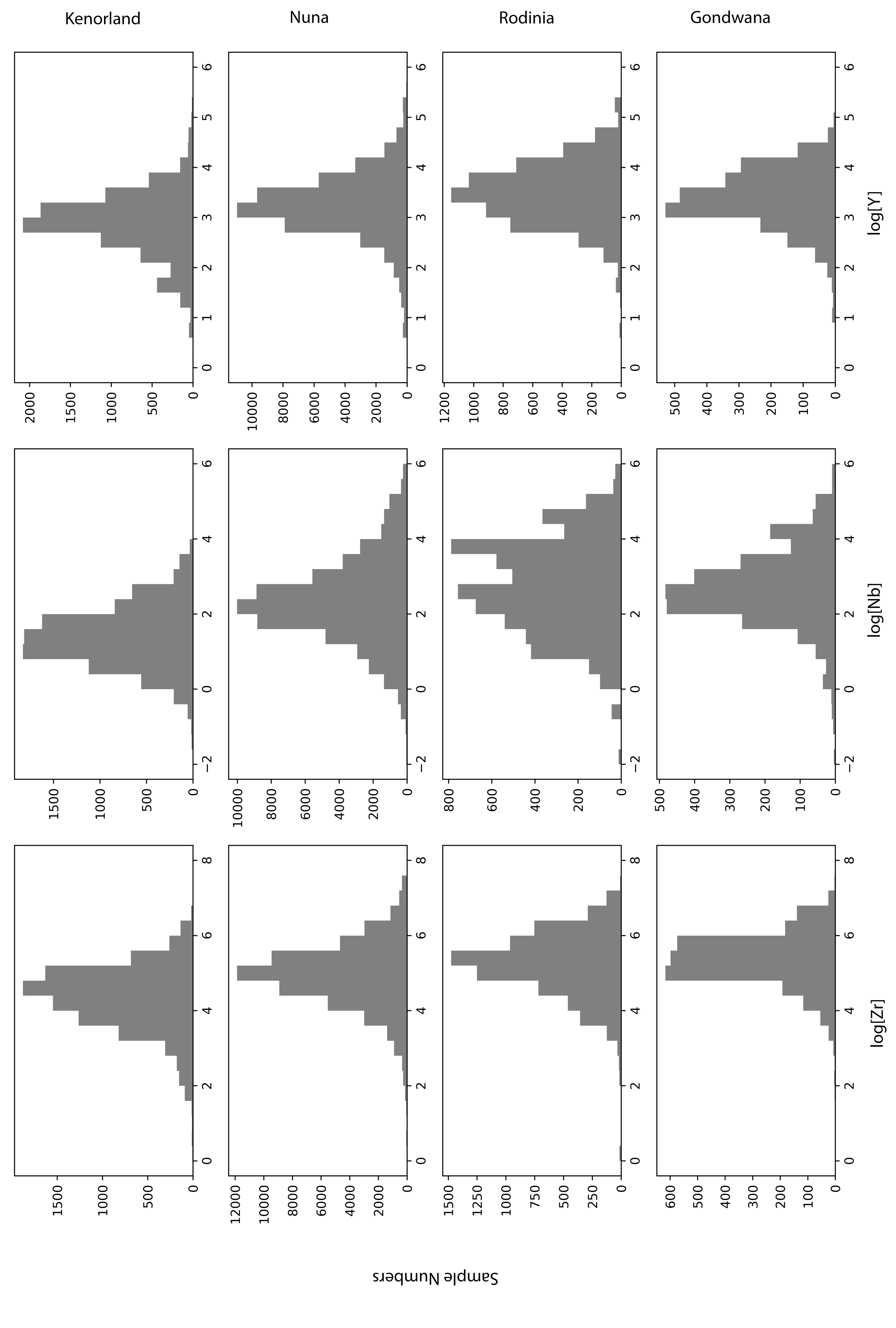

Supplement: Supplementary file 1 — Supplementary Information [file 41467_2017_2095_MOESM1_ESM.pdf]
